# Histopathological Evaluation of Bioactive Glass Wound Sites in a Swine Model

**DOI:** 10.3390/bioengineering13020200

**Published:** 2026-02-11

**Authors:** Daniel A. Rabin, Aneeq S. Chaudhry, Tarifa H. Adam, Katherine Kozlowski, Marlynn P. Lopez, Tiffany Kim, Spencer Green, Robert D. Galiano, Gregory C. Manista, Donald W. Buck, Steven Jung

**Affiliations:** 1 Division of Plastic & Reconstructive Surgery, Northwestern University Feinberg School of Medicine, Chicago, IL 60187, USA; daniel.rabin@northwestern.edu (D.A.R.); aneeq.chaudhry@northwestern.edu (A.S.C.); tarifa.adam@northwestern.edu (T.H.A.); katherine.kozlowski@northwestern.edu (K.K.); marlynn.lopez@northwestern.edu (M.P.L.); tiffany.kim@my.rfums.org (T.K.); spencergreen@creighton.edu (S.G.); robert.galiano@nm.org (R.D.G.); 2 Engineered Tissue Solutions (ETS), Rolla, MO 65401, USA; dbuck@etswoundcare.com; 3 Mo Sci LLC., Rolla, MO 65401, USA; sjung@mo-sci.com

**Keywords:** wound healing, chronic wounds, bioactive glass matrix, animal research, wound matrix

## Abstract

Chronic wounds, including diabetic foot ulcers (DFUs), venous leg ulcers (VLUs), and pressure injuries, remain a major global health burden and contribute substantially to Medicare spending. Because traditional wound dressings fail to address the dynamic microenvironment of chronic wounds, bioactive materials that modulate inflammation and support tissue regeneration are needed. In this study, we evaluated the tissue response to our borate-based bioactive glass fiber matrix (BBGFM) designed to overcome limitations of existing fibrous wound dressings. Two *Sus scrofa domesticus* underwent creation of twelve 5 × 5 cm subcutaneous pockets each, which were treated with BBGFM at three thicknesses (25%, 50%, and 100%) or left untreated as controls. One animal was euthanized at three weeks and the other at six weeks for gross and histopathological evaluation of all wound sites. BBGFM-treated pockets demonstrated a dose-dependent increase in inflammation at three weeks that diminished by six weeks. Enhanced neovascularization and collagen matrix deposition were also seen at both time points. Collagen maturity increased across all groups by six weeks, and residual BBGFM correlated with initial implant thickness. These findings indicate that BBGFM promotes a controlled inflammatory response and supports neovascularization and matrix remodeling in a dose-dependent manner, suggesting its potential as an effective bioactive wound matrix.

## 1. Introduction

Chronic wounds, including diabetic foot ulcers (DFUs), venous leg ulcers (VLUs), and pressure injuries, pose a significant and escalating global healthcare challenge. These wounds are marked by persistent inflammation, impaired re-epithelialization, and heightened susceptibility to infection, often resulting in delayed healing, limb amputation, and increased mortality [1,2,3]. In the United States alone, chronic wounds affect approximately 8.2 million Medicare beneficiaries annually, costing up to USD 96.8 billion per year [4,5]. This burden is expected to grow due to aging populations and rising rates of diabetes and vascular disease, highlighting an urgent need for more effective and accessible wound care strategies [6].

Wound healing is a highly coordinated biologic process involving four overlapping, interdependent phases [7]. It begins with hemostasis, which occurs immediately after injury and typically lasts 12–24 h [7]. During this phase, local vasoconstriction occurs and platelets are activated upon contact with exposed collagen, leading to granule release and initiation of the coagulation cascade. This response sets the stage for the inflammatory phase, which begins within hours of injury and can last up to a week. Pro-inflammatory cytokines such as IL-1β, IL-6, and TNF-α are released, promoting recruitment of neutrophils, followed by macrophages. Neutrophils eliminate pathogens through the oxidative burst and phagocytosis, while macrophages, initially in their M1 pro-inflammatory state, degrade extracellular matrix and debris through the secretion of matrix metalloproteinases (MMPs) [8]. As healing progresses, macrophages transition into their M2 anti-inflammatory phenotype. M2 macrophages support tissue repair by releasing VEGF, promoting angiogenesis and stimulating fibroblasts to produce collagen [8]. The proliferative phase, which overlaps with inflammation and can last several weeks, involves the formation of granulation tissue, re-epithelialization, neovascularization, and active immunomodulation [9]. Activated fibroblasts play a central role by synthesizing extracellular matrix components and facilitating wound contraction by the action of myofibroblasts [10]. A critical aspect of this phase is angiogenesis, as newly formed blood vessels supply the oxygen and nutrients necessary for sustained tissue repair. The final stage of healing is remodeling. Remodeling can extend up to a year following injury [7]. During this phase, type III collagen initially deposited in the wound bed is gradually replaced by more mechanically robust type I collagen, improving the mechanical strength and durability of the regenerated tissue. Aberrations at any stage of the wound healing process lead to chronic wounds or pathologic scarring. 

Traditional wound dressings often inadequately address the complex, evolving microenvironment of chronic wounds [11]. In recent decades, bioactive wound matrices have emerged as promising alternatives [12,13,14]. These materials influence key cellular behaviors, particularly those involved in inflammation, proliferation, and tissue remodeling. Among these, fibrous bioactive materials stand out for their structural and mechanical resemblance to native extracellular matrices [15,16]. Their porous architecture and adaptable material composition, ranging from organic to inorganic elements, create a favorable biochemical and physical environment for optimal healing [17,18]. Bioglass-based materials have garnered particular interest within this category. Early iterations, such as silicate-based bioglass, are beneficial for treating skin ulcers and soft tissue wounds [19]. More recently, borate-based bioglass has garnered increased recognition. Borate plays an important role in wound healing, namely, through enhancement of keratinocyte migration, antimicrobial activity, and regulation of collagen, protein, and proteoglycan deposition [20,21,22]. Additionally, borate offers greater biodegradability and enhanced bioactivity [23]. Ultimately, it converts into hyaluronic acid-like substances to further support the healing process [24].

Building on these advances, Mirragen Advanced Wound Matrix (ETS Wound Care; Rolla, MO, USA) is a borate-based bioactive glass fiber matrix (BBGFM) composed entirely of naturally occurring elements [22,25]. This matrix consists of a combination of fibers and beads with a target composition of 53B_2_O_3_–20CaO–12K_2_O–6Na_2_O–5MgO–4P_2_O_5_ mol% that degrade through hydrolysis over 1–2 weeks (fibers) and 3 or more weeks (beads), respectively [25]. This staggered degradation profile sustains a regenerative environment to facilitate high-quality tissue formation across various wound types. The BBGFM serves as a scaffold for tightly-packed collagen deposition, reinforcing tissue strength and enabling it to endure physiologic stress. It also promotes robust angiogenesis, resulting in highly vascularized and well-organized granulation tissue. Due to its acellular composition, BBGFM demonstrates excellent biocompatibility and low immunogenicity, which further enhances its therapeutic potential.

The BBGFM is indicated by the United States Food and Drug Administration (FDA) for a broad spectrum of wounds, including partial- and full-thickness wounds, pressure injuries, venous and diabetic ulcers, chronic vascular ulcers, surgical wounds (including donor sites, grafts, Mohs and post-laser procedures, podiatric wounds, and wound dehiscence), trauma-related wounds such as abrasions, lacerations, burns, and skin tears, as well as draining wounds [26]. Using a porcine wound model, we performed histological evaluations at two distinct time points to compare cellular and tissue responses in subcutaneous pockets treated with and without BBGFM. These evaluations focused on levels of inflammation, collagen matrix deposition, and neovascularization.

## 2. Materials and Methods

Two *Sus scrofa domesticus* pigs were used to histologically assess wound sites treated with BBGFM. In vivo work and tissue collection was performed at Cooper University Health Care (Camden, NJ, USA). The protocol was approved by the Cooper University Health Care Animal Care Committee, approval number 23-019. Each animal underwent a pre-procedural physical examination and was weighed prior to anesthesia induction. Blood was collected for clinical pathology and the animals were transferred to the surgical suite. Animals were intubated, mechanically ventilated, and maintained under general anesthesia with isoflurane. An intravenous electrolyte infusion and intramuscular antibiotic prophylaxis were administered. A rectal temperature probe was placed, and electrocardiogram, arterial blood pressure, and oxygen saturation were continuously monitored. Any abnormalities were addressed with appropriate clinical interventions. 

On day zero, twelve 5 × 5 cm subcutaneous defects (pockets) were created on the dorsum of each animal. Prior to beginning the procedure, the BBGFM wound matrix was manually sectioned using a scalpel to achieve the desired thickness of the manufactured material. Nine BBGFMs of various thicknesses (100% thickness (*n* = 3), 50% thickness (*n* = 3), and 25% thickness (*n* = 3)) were placed inside the defects. Incisions were then closed and recovered. Three control pockets with no material were created in each animal. 

Animals were housed for either three or six weeks according to institutional housing SOPs. Wounds were photographed three times weekly. One animal was euthanized at 3 weeks and the other at 6 weeks. A limited necropsy, which consisted of excising and gross examination of the target tissue (pocket with surrounding skin), was performed after euthanasia. Histopathological evaluation included 24 wound sites from two pigs. Wound sites intended for processing and histopathological evaluation were shipped in 10% NBF to StageBio (Frederick, MD, USA). Samples from the 3-week cohort were processed in paraffin or resin depending on degradation, and the 6-week samples were processed similarly based on preliminary findings. Upon receipt at StageBio, tissues were trimmed to sample a single cross section of skin, subcutis, and deeper tissues by cutting parallel and ~1 cm ventral to the healed skin incision. Tissues were submitted for oversized paraffin processing and stained with Hematoxylin and Eosin (H&E), Herovici’s, and immunohistochemically labeled for CD31 (chromogenic).

All resulting slides were evaluated in a blinded fashion by the study pathologist (D.G.). Slides were screened for wound healing response, and evaluated for the following parameters: inflammation (overall inflammation, neutrophils, eosinophils, lymphocytes, plasma cells, macrophages, multinucleated giant cells), IHC CD31, neovascularization, fibrosis, collagen maturity, pseudocyst, hemorrhage, pigment, macrophage cytoplasm, residual BBGFM presence, and foreign material presence. Slides were scored according to the grading scales depicted in Table 1. Scores from all slides in each experimental group were then averaged and reported.

### Histomorphometric Analysis

H&E-stained slide images were marked with the boundaries of visible scar tissue prior to analysis by the study pathologist. The Line (L) Tool in ImagePro Plus 7 was used to measure the linear thickness of the scar tissue perpendicular to the epithelial surface 1–2 mm from the left edge of the marked region, measuring every 2 mm from left to right, stopping 1–2 mm from the rightmost edge. Where scar tissue was not contiguous and therefore not present at a 2 mm interval, no measurement was made.

## 3. Results

Overall, the microscopic appearance of the surgically created pockets were similar morphologies at both time points as characterized by neovascularization, fibrosis, and inflammatory cell infiltrates. However, there were differences in the intensity of these findings in the BBGFM implant defect sites versus the control defect sites. Residual BBGFM was noted in all BBGFM-treated defect sites at both time points, apparent microscopically as round or irregularly shaped particulates with basophilic-staining in the H&E sections. Generally, there was more residual BBGFM material noted in wounds treated with a larger thickness matrix.

### 3.1. Inflammation

**Figure 1:**
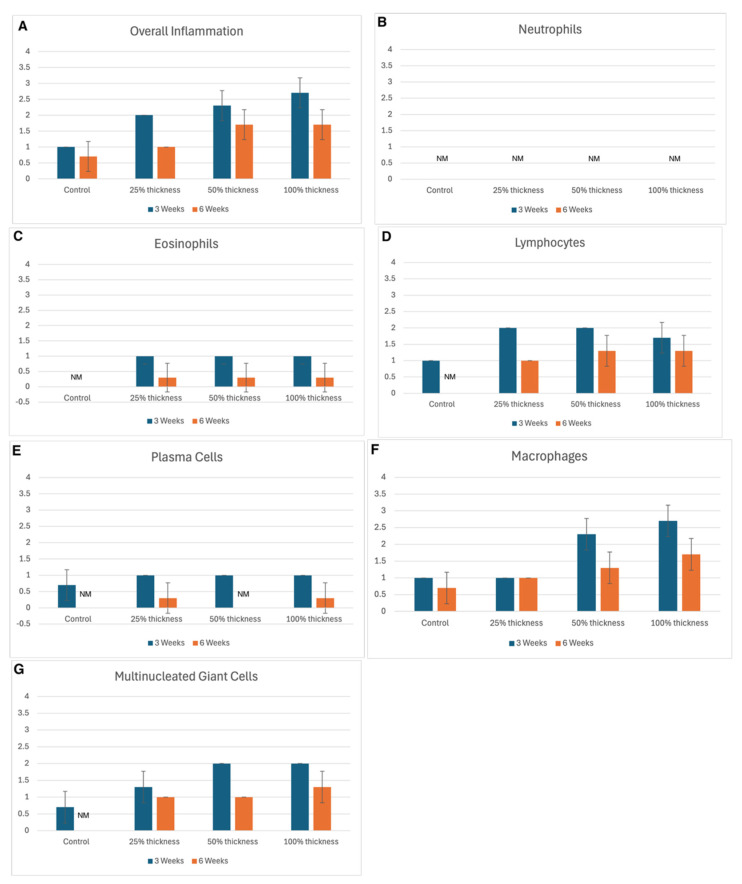
(**A**–**G**) Inflammation and inflammatory cell types in controls versus BBGFM at time points 3 weeks and 6 weeks. Graded on a scale of 0–4, as depicted in Table 1B,C for respective cell types (NM = not measured). Bars represent mean values; error bars indicate standard deviation.

At the 3-week time point, overall inflammation was negligible in the untreated control group, whereas BBGFM-treated groups exhibited a dose-dependent increase in overall inflammation (Figure 1A). This increase was associated with the presence of residual BBGFM and was characterized predominantly by macrophage infiltration (Figure 1F). By 6 weeks, the intensity of overall inflammation in BBGFM-treated groups had decreased compared with the earlier time point; however, inflammatory cells remained more abundant in the 50% and 100% thickness groups than in the untreated control group (Figure 1A). An overview of the observed inflammation for each BBGFM thickness at both time points is shown in Figure 1A–G.

### 3.2. Neovascularization/Fibrosis

**Figure 2:**
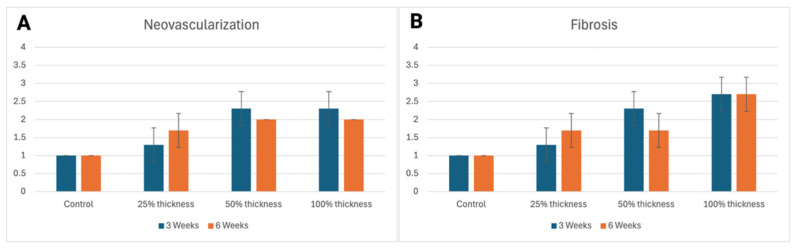
(**A**,**B**) Neovascularization (**A**) and fibrosis (**B**) in controls versus BBGFM at time points 3 weeks and 6 weeks, respectively. Graded on a scale from 0 to 4, as depicted in Table 1D. Bars represent mean values; error bars indicate standard deviation.

The presence of new collagen matrix deposition was accompanied by the presence of neovascularization at both time points, marked by a greater intensity of neovascularization and fibrosis in the BBGFM group relative to the control group. This increase was seen in a dose-dependent fashion for both parameters, as seen in Figure 2A,B.

### 3.3. Herovici Stains

**Figure 3:**
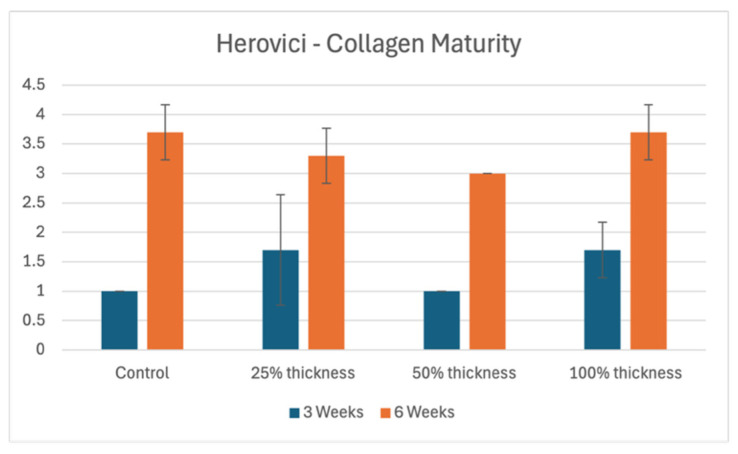
Herovici stains for collagen maturity in controls versus BBGFM at time points 3 weeks and 6 weeks. Graded on a scale from 0 to 4, as depicted in Table 1A. Bars represent mean values; error bars indicate standard deviation.

**Figure 4:**
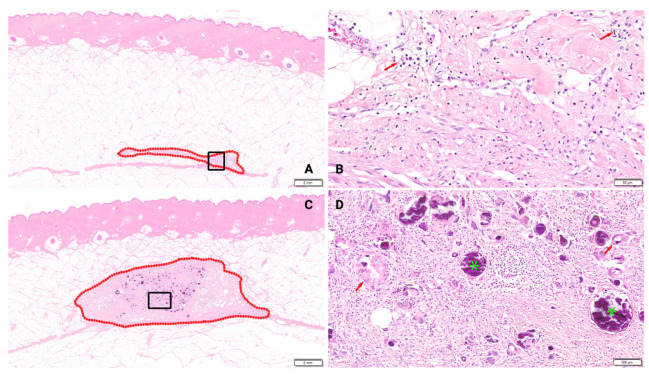
(**A**–**D**) Histological sections depicting collagen maturity at 3 weeks. Graded on a scale from 0 to 4, as depicted in Table 1A. (**A**)—Animal 1, Wound site 1 (H&E), Control, 3 weeks. Host tissue response in surgically created pocket outlined in red. Fibrosis score = Grade 1. Close up of the region within the rectangle presented in (**B**). (**B**)—Animal 1, Wound site 1 (H&E), Control, 3 weeks. Close up of Grade 1 fibrosis (eosinophilic matrix), neovascularization, and inflammatory cell infiltrates (at arrows). (**C**)—Animal 1, Wound site 7 (H&E), BBGFM wound matrix (100% thickness), 3 weeks. Host tissue response in surgically created pocket outlined in red. Fibrosis score = Grade 3. Close up of the region within the rectangle presented in (**D**). (**D**)—Animal 1, Wound site 7 (H&E), BBGFM (100%), 3 weeks. Close up of Grade 3 fibrosis (eosinophilic matrix), neovascularization, and inflammatory cell infiltrates (predominated by macrophages, lymphocytes, and multinucleated giant cells). Note residual BBGFM material (example at green asterisks) and multinucleated giant cells (example at arrows).

**Figure 5:**
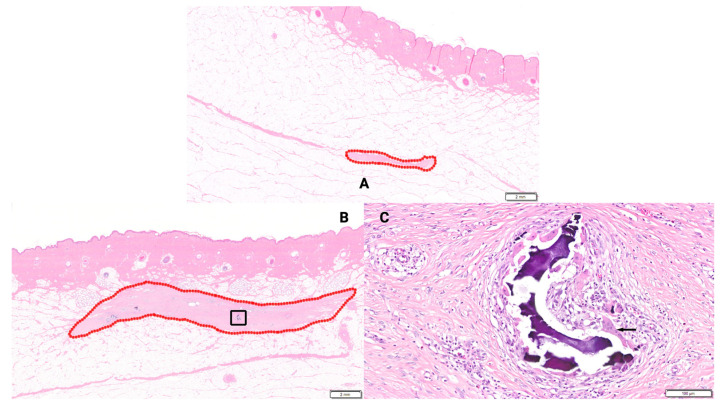
(**A**–**C**) Histological sections depicting collagen maturity at 6 weeks. Graded on a scale from 0 to 4, as depicted in Table 1A. (**A**)—Animal 2, Wound site 1 (H&E), Control, 6 weeks. Host tissue response in surgically created pocket outlined in red. Fibrosis score = Grade 1. (**B**)—Animal 2, Wound site 7 (H&E), BBGFM wound matrix (100% thickness), 6 weeks. Host tissue response in surgically created pocket outlined in red. Fibrosis score = Grade 3. Close up of the region within the rectangle presented in (**C**). (**C**)—Animal 2, Wound site 7 (H&E), BBGFM wound matrix (100% thickness), 6 weeks. Close up of Grade 3 fibrosis (eosinophilic matrix), with residual BBGFM material associated with inflammatory cell infiltrates. Note multinucleated giant cells (example at arrow).

There were no consistent patterns in collagen maturity at the 3-week time point, represented by variable maturity in control groups and all three BBGFM-treated groups (Figure 3 and Figure 4). At the later time point, defects had higher collagen maturity ratings in all four groups as expected, although no clear trends relative to BBGFM thickness were observed (Figure 3 and Figure 5).

### 3.4. Immunohistochemistry Evaluation—Semi-Quantitative for CD31

**Figure 6:**
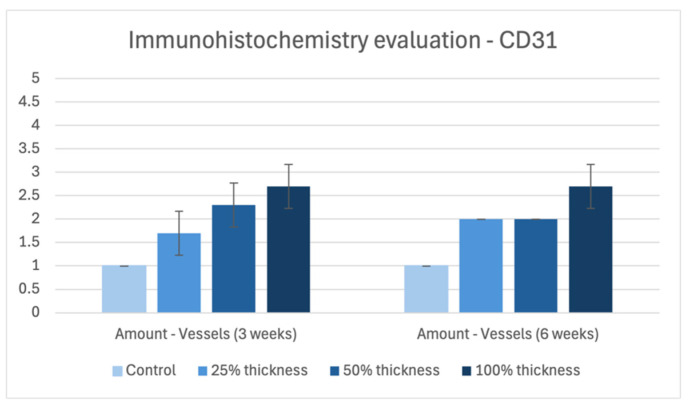
Immunohistochemistry evaluation of CD31 in controls versus BBGFM at time points 3 weeks and 6 weeks, respectively. Graded on a scale from 0 to 5, as depicted in Table 1E. Bars represent mean values; error bars indicate standard deviation.

**Figure 7:**
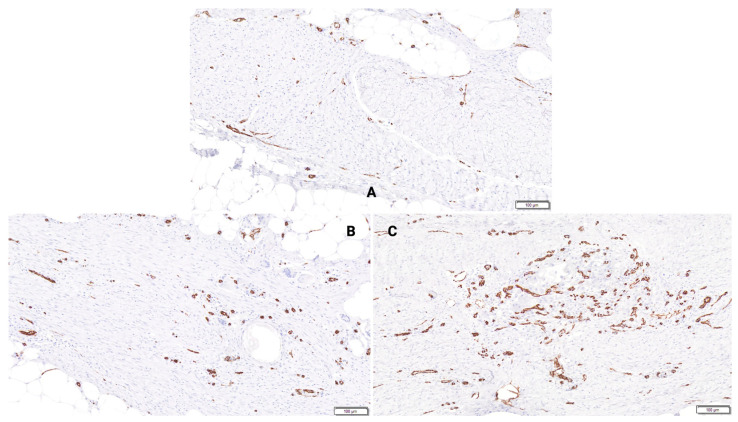
Immunohistochemistry for detection of CD31 at 6 weeks. Graded on a scale from 0 to 5, as depicted in Table 1E. (**A**)—Animal 2, Wound site 1 (CD31), Control, 6 weeks. Amount score = Grade 1. (**B**)—Animal 2, Wound site 2 (CD31), BBGFM wound matrix (25% thickness), 6 weeks. Amount score = Grade 2. (**C**)—Animal 2, Wound site 4 (CD31), BBGFM wound matrix (100% thickness), 6 weeks. Amount score = Grade 3.

At both time points, CD31 expression increased in a BBGFM thickness-dependent fashion. However, CD31 ratings remained constant for each thickness between the two time points (Figure 6 and Figure 7).

### 3.5. Histomorphometry Evaluation

**Figure 8:**
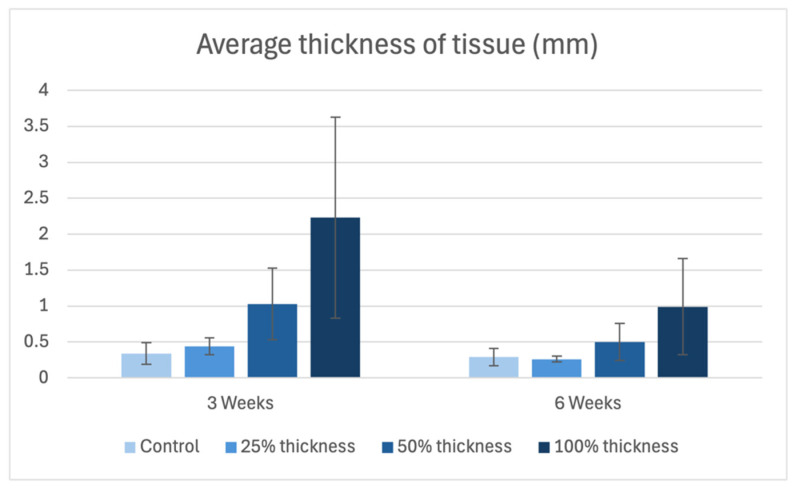
Average thickness of tissue (mm) in controls versus BBGFM at time points 3 weeks and 6 weeks. Bars represent mean values; error bars indicate standard deviation.

Healed tissue thickness demonstrated a dose-dependent increase at both time points. Additionally, it is noteworthy that there was a reduction in tissue thickness at the latter time point compared to the early time point (Figure 8).

## 4. Discussion

Aberrant wound healing presents a major clinical and economic burden, with chronic wounds contributing to an estimated USD 96.8 billion in annual healthcare costs [4,5]. Advances in biomaterials have permitted the development of synthetic matrices that can be tailored to augment biological processes relevant to healing while simultaneously addressing microbial presence using non-antibiotic approaches. 

### 4.1. Inflammation

A hallmark of chronic wounds is dysregulated inflammation [27,28]. In our model, BBGFM-treated wounds exhibited a thickness-dependent increase in inflammation at 3 weeks, characterized predominantly by macrophages with fewer lymphocytes and multinucleated giant cells. This early inflammatory response likely reflects the bioactivity and partial degradation of the material. By 6 weeks, inflammation had subsided across all treatment groups, indicating resolution of the initial immune response and supporting both the biocompatibility and appropriate degradation profile of the BBGFM. This temporal pattern suggests that the matrix elicits a beneficial, self-limiting inflammatory cascade essential for wound healing without promoting chronic inflammation. Notably, neutrophils were absent at both time points, indicating that the inflammation observed was reparative rather than pathologic, driven by macrophages and multinucleated giant cells. However, further characterization of the inflammatory milieu would be speculative, as macrophage polarization was not assessed. Future studies incorporating more granular assessments of inflammatory characteristics, particularly macrophage polarization, are necessary to verify that the BBGFM matrix induces a beneficial, pro-resolutionary inflammation.

### 4.2. Neovascularization

Impaired neovascularization is a known barrier to wound healing [29,30]. BBGFM-treated wounds demonstrated consistent increases in neovascularization at both time points in a dose-dependent manner, supported by both histological scoring and CD31 immunohistochemistry. These results underscore BBGFM’s ability to stimulate angiogenesis, a critical step for delivering nutrients, oxygen, and immune cells to the healing site, and suggest that its porous structure and degradation products facilitate vascular in-growth.

### 4.3. Collagen Deposition and Maturity

Fibroblast-driven collagen deposition is essential for wound strength and remodeling [10,31]. BBGFM-treated wounds exhibited increased fibrosis at both time points, particularly in the 100% thickness BBGFM group. The newly deposited collagen displayed variable maturity at 3 weeks, but showed maturation by 6 weeks, aligning with the expected wound healing timeline. Although differences in collagen maturity between groups were modest at 6 weeks, the trend supports BBGFM’s role in advancing wound matrix organization. Future long-term studies will be needed to further evaluate collagen remodeling and the mature-to-immature collagen ratio at later time points, which are critical metrics of durable wound repair [10].

### 4.4. Scar Thickness and Vascular Density

Histomorphometric analysis showed that BBGFM-treated wounds had greater scar thickness in a dose-dependent fashion at both time points. Interestingly, all groups, including controls, showed reduced scar thickness by 6 weeks, likely reflecting natural wound contraction and remodeling. Vascular density, measured by CD31-positive vessels per mm^2^, was trended higher in BBGFM-treated wounds, especially in the 100% thickness group, further validating the matrix’s pro-angiogenic effects [32].

### 4.5. Biodegradation and Material Persistence

Residual BBGFM was visible microscopically at both time points, with amounts correlating to the initial dose. Importantly, decreasing inflammation over time suggests that BBGFM degrades in a biologically appropriate timeframe. These results are consistent with the degradation and inflammatory profiles of other commonly-used skin substitutes, including Integra Dermal Regeneration Template, MatriStem Porcine small intestinal submucosa, AlloDerm Human acellular dermal matrices, and NovoSorb^®^ BTM Synthetic biodegradable dermal matrices, among many others [33,34,35,36]. Each of these matrices and dressings degrade over weeks to months, which were strategically designed to have longitudinal effects in the wound bed to coordinate a longer-lasting improved wound healing outcome [33,34,35,36]. Similarly, a concordant inflammatory response is unavoidable due to the presence of foreign material. However, the macrophage-dominant inflammation maintained longitudinally seen in histological assessments of these matrices represents a form of pro-resolutionary inflammation rather than a problematic neutrophil-dominant inflammation [37]. Future investigations should determine the optimal BBGFM thickness that maximizes regenerative effects while minimizing persistent inflammation.

### 4.6. Limitations

This study is limited by the small sample size. Based on the pilot design, only two animals were used, and twelve wounds in each animal were created. Despite clear trends in differences between the BBGFM and the control groups, the small sample size limits the feasibility of obtaining inferential statistics, limiting the strength of our conclusions. Future studies with larger sample sizes of animals and/or humans will be beneficial in further elucidating the true differences between the BBGFM and controls. Additionally, this study was performed in swine. Swine represent good models of comparisons for human wound healing; however, they are not completely analogous. For this reason, human experimentation is necessary to validate these results following future larger-scale animal trials. 

## 5. Conclusions

Our findings demonstrate that BBGFM enhances key aspects of wound healing, particularly the inflammatory response, neovascularization, and collagen deposition, in a dose-dependent manner. The BBGFM’s degradation kinetics support a controlled inflammatory phase followed by tissue regeneration, aligning with the physiological wound healing timeline. These preliminary results position BBGFM as a promising candidate for future clinical application in managing complex wounds. Though not yet a replacement, future larger animal and clinical studies in support of these findings will validate the BBGFM as an adjunct biomaterial to improve wound healing outcomes.

## Figures and Tables

**Table 1:** **Grading scales for histology and immunohistochemistry evaluation.** (**A**) outlines the grading scale for extent of collagen deposition. (**B**) details the grading scale for inflammatory cell types seen under microscopy. (**C**) presents the grading scale for multinucleated giant cells. (**D**) presents the general scoring of all other parameters, including overall inflammation, neovascularization, fibrosis, pseudocyst, hemorrhage, pigment, and macrophage cytoplasm. (**E**) depicts the grading scale for detection of CD31 on immunohistochemistry.

**(A) Extent of Collagen Deposition/Maturity**	**Score**
No collagen deposition	0
Majority of wound composed of immature collagen fibers (~1% to <50%)	1
Approximately equal amounts of immature and mature collagen fibers within wound	2
The majority of wound composed of mature collagen fibers (~51% to <95%)	3
Essentially all of wound composed of mature collagen fibers (~>95%)	4
**(B) Neutrophils, Eosinophils, Lymphocytes, Plasma Cells, Macrophages**	**Score**
Absent	0
Rare, 1–5/hpf	1
6–10/hpf	2
Moderate, heavy infiltrates	3
Packed	4
**(C) Multinucleated Giant Cells**	**Score**
Absent	0
Rare, 1–2/hpf	1
3–5/hpf	2
Moderate, heavy infiltrates	3
Sheets	4
**(D) General Scoring**	**Score**
Absent	0
Minimal	1
Mild	2
Moderate	3
Marked/Severe	4
**(E) Amount**	**Score**
Negative, no reactivity	0
Very rare, estimated as less than 5%	1
Rare, estimated as 5–25%	2
Occasional, estimated as 26–50%	3
Frequent, estimated as 51–75%	4
Very frequent, estimated as 76–100%	5

## Data Availability

The original contributions presented in this study are included in this article; further inquiries can be directed to the corresponding author.

## Abbreviations

The following abbreviations are used in this manuscript:

DFU	Diabetic Foot Ulcer
VLU	Venous Leg Ulcer
BBGFM	Borate-Based Bioactive Glass Fiber Matrix
MMP	Matrix Metalloproteinase
IL	Interleukin
TNF	Tissue Necrosis Factor
VEGF	Vascular Endothelial Growth Factor
FDA	Food and Drug Administration
SOP	Standard Operating Procedure
NBF	Neutral Buffered Formalin
H&E	Hematoxylin and Eosin
IHC	Immunohistochemistry
